# A new species of *Nanorana* Günther, 1896 (Anura, Dicroglossidae) from Yunnan, China

**DOI:** 10.3897/zookeys.1048.65620

**Published:** 2021-07-07

**Authors:** Shuo Liu, Peisong Zhang, Dingqi Rao

**Affiliations:** 1 Kunming Natural History Museum of Zoology, Kunming Institute of Zoology, the Chinese Academy of Sciences, 32 Jiaochang Donglu, Kunming, Yunnan 650223, China Kunming Natural History Museum of Zoology Kunming China; 2 Research Institute of Xishuangbanna National Nature Reserve, No. 6 North Galan Road, Jinghong, Yunnan 666100, China Research Institute of Xishuangbanna National Nature Reserve Jinghong China; 3 Kunming Institute of Zoology, the Chinese Academy of Sciences, No. 17 Longxin Road, Kunming, Yunnan 650201, China Kunming Institute of Zoology Kunming China

**Keywords:** 12S, 16S, morphology, phylogeny, spiny frog, systematic, taxonomy

## Abstract

A new species of *Nanorana* Günther, 1896 is described from Yunnan Province, China, based on morphological and molecular evidence. Morphologically, *Nanorana xuelinensis***sp. nov.** is distinguished from its congeners by a combination of the following diagnostic characters: body size large; adult males with keratinized spines on chest, belly, lateral body, posterior dorsum, buttocks, outer side of the fore limbs, the inner metacarpal tubercle, fingers I and II, and upper eyelids; no spines on the inner side of the lower and upper arm; forelimbs strongly hypertrophied in adult males; anterior dorsum skin smooth; dorsolateral folds absent; finger I longer than finger II; webbing deeply incurved between tips of toes; present outer metacarpal tubercle and absent outer metatarsal tubercle. The new species is separated from all other congeners by uncorrected genetic distances ranging from 5.2% to 7.3% based on mitochondrial 16S rRNA gene and ranging from 3.9% to 7.6% based on mitochondrial 12S rRNA gene.

## Introduction

The tribe Paini is a widespread, complex taxon, and there are many different views on the classification of this taxon. Dubois (1992) first proposed the tribe Paini to include the genera *Paa* Dubois, 1975 and *Chaparana* Bourret, 1939. Roelants et al. (2004) suggested that *Nanorana* Günther, 1896 is imbedded within *Paa* on the basis of molecular data. Jiang et al. (2005) presented that *Quasipaa* Dubois, 1992 is a distinct genus in the tribe Paini. Chen et al. (2005) placed *Chaparana*, *Paa*, and *Nanorana* into *Nanorana* on the basis that *Paa* is paraphyletic with respect to *Nanorana* and *Chaparana*. Ohler and Dubois (2006) described two new genera in the tribe Paini, namely *Allopaa* Ohler & Dubois, 2006 and *Chrysopaa* Ohler & Dubois, 2006. Che et al. (2010, 2020) considered that the high elevation species of *Nanorana* represent dwarfed and degraded ones derived from lower elevation *Paa* on the basis of evidence of molecular phylogeny. Dubois et al. (2021) presented a different classification of the tribe Paini that included more genera, namely *Chaparana*, *Diplopaa* Dubois, Ohler & Pyron, 2021, *Feirana* Dubois, 1992, *Gynandropaa* Dubois, 1992, *Nanorana*, *Ombropaa* Dubois, Ohler & Pyron, 2021, and *Paa*.

To reduce confusion, we currently use the classification system on the “Amphibian Species of the World” website (Frost 2021). In this classification system, the genus *Nanorana* now contains 30 species (Frost 2021), of which 21 species were recorded in China (AmphibiaChina 2021).

During a field survey in Yunnan, China in 2019, some specimens of the genus *Nanorana* were collected. Morphological and molecular analyses indicated that these frogs were distinctive, differing from all known species of genus *Nanorana*. Therefore, we described them here as a new species.

## Materials and methods

### Sample collection

Specimens were collected by hand from Lancang County, Yunnan, China, euthanized, tissue samples taken, then preserved in 75% ethanol. Tissue samples were taken from liver and placed in 99% ethanol and subsequently stored at −80 °C. All specimens were deposited at Kunming Natural History Museum of Zoology, Kunming Institute of Zoology, the Chinese Academy of Sciences (**KIZ**).

### Laboratory methods

Genomic DNA extracted from 99% ethanol-preserved liver tissues, using DNA extraction kit from Beijing Dingguo Changsheng Biotechnology Co. Ltd. Two mitochondrial genes, 12S and 16S, were amplified. Primers used for 12S were FS01: 5'-AACGCTAAGATGAACCCTAAAAAGTTCT-3' and R16: 5'-ATAGTGGGGTATCTAATCCCAGTTTGTTTT-3' (Qi et al. 2019) and for 16S were 16Sar: 5' -CGCCTGTTTACCAAAAACAT-3' and 16Sbr: 5'-CCGGTYTGAACTCAGATCAYGT-3' (Palumbi et al. 1991). PCR conditions followed Qi et al. (2019). Amplifications were processed with the cycling conditions that initial denaturing step at 94 °C for 5 min, 35 cycles of denaturing at 94 °C for 30 sec, annealing at 55 °C for 30 sec and extending at 72 °C for 1 min, and final extending step at 72 °C for 5 min. PCR products were isolated through electrophoresis using 1% agarose gels, and further purified using Millipore Microcon Kits. Purified PCR products were sequenced by Davis Sequencing using BigDye terminator 3.1 and sequences were edited and manually managed using SeqMan in Lasergene 7.1 (DNASTAR Inc., Madison, WI, USA) and MEGA X (Kumar et al. 2018). All sequences were deposited in GenBank (Table 1).

**Table 1.** Information of samples used in molecular analysis.

Species name	Locality	Specimen voucher	12S	16S	Rag1	Rhod	Tyr
*Nanorana aenea*	Sa Pa, Lao Cai, Vietnam	ROM37984	EU979693	EU979830	HM163609	EU979895	EU979986
*Nanorana aenea*	Sa Pa, Lao Cai, Vietnam	MNHN 1999.5818	AY880456	AY880443	–	–	–
*Nanorana blanfordii*	Yatung, Tibet, China	SYNU-1507011	MH315954	MH315963	–	–	–
*Nanorana chayuensis*	Zayü, Tibet, China	SYNU-XZ64	EU979709	DQ118509	–	EU979853	EU979944
*Nanorana chayuensis*	Zayü, Tibet, China	SYNU-XZ67	EU979708	DQ118510	–	EU979852	EU979943
*Nanorana conaensis*	Cona, Tibet, China	KIZ-YP152	EU979703	EU979834	–	EU979874	EU979965
*Nanorana liebigii*	Janakpur, Nepal	A17_12_NME	MN011989	MN012104	MN032528	MN012368	MN012518
*Nanorana liebigii*	Janakpur, Nepal	R18_12_NME	–	MN012105	MN032529	MN012369	MN012519
*Nanorana maculosa*	Jingdong, Yunnan, China	YNU-HU2002308	EU979706	EU979835	–	EU979859	EU979950
*Nanorana maculosa*	Jingdong, Yunnan, China	YNU-HU2002322	EU979707	DQ118512	–	EU979860	EU979951
*Nanorana medogensis*	Medôg, Tibet, China	SYNU-XZ35	EU979705	DQ118506	–	EU979862	EU979953
*Nanorana medogensis*	Medôg, Tibet, China	SYNU-XZ75	EU979704	DQ118507	–	EU979861	EU979952
*Nanorana parkeri*	–	N7_06_NME	MN012006	MN012126	MN032549	MN012391	MN012540
*Nanorana parkeri*	Dangxiong, Tibet, China	–	KP317482	KP317482	–	–	–
*Nanorana phrynoides*	Yimen, Yunnan, China	YNU-HU20024012	EU979686	EU979825	–	EU979877	EU979968
*Nanorana pleskei*	–	KQ47_14_NME	MN012019	MN012156	MN032562	MN012422	MN012570
*Nanorana pleskei*	Shiqu, Sichuan, China	CIB20080515-1	HQ324232	HQ324232	–	–	–
*Nanorana polunini*	Pangum, Nepal	K1553	–	KR827957	–	–	–
*Nanorana quadranus*	An, Sichuan, China	SCUM20030031GP	EU979694	EU979831	–	EU979886	EU979977
*Nanorana quadranus*	Maowen, Sichuan, China	SCUM20045195CJ	EU979695	DQ118514	–	EU979887	EU979978
*Nanorana rostandi*	Kyirong, Tibet, China	SYNU-1507058	MH315955	MH315964	–	–	–
*Nanorana sichuanensis*	Huili, Sichuan, China	SCUM20030091GP	EU979685	EU979824	–	EU979880	EU979971
*Nanorana taihangnica*	Jiyuan, Henan, China	KIZ-HN0709001	EU979724	EU979842	–	EU979893	EU979984
*Nanorana taihangnica*	Jiyuan, Henan, China	KIZ-HN0709002	EU979725	EU979843	–	EU979894	EU979985
*Nanorana unculuanus*	Jingdong, Yunnan, China	YNU-HU2002502601	EU979699	DQ118490	–	DQ458262	DQ458277
*Nanorana unculuanus*	Jingdong, Yunnan, China	YNU-HU2002502702	EU979700	DQ118491	HM163585	EU979865	EU979956
*Nanorana ventripunctata*	–	SH050538_NME	MN012066	MN012208	MN032610	MN012478	MN012626
*Nanorana ventripunctata*	Xianggelila, Yunnan, China	SCUM045887WD	EU979717	DQ118501	HM163585	EU979868	EU979959
*Nanorana yunnanensis*	Yongde, Yunnan, China	YNU-HU20011102	EU979691	EU979829	–	EU979884	EU979975
*Nanorana zhaoermii*	Lhünzê, Tibet, China	SYNU-1706049	MH315947	MH315956	–	–	–
*Nanorana zhaoermii*	Lhünzê, Tibet, China	SYNU-1706058	MH315948	MH315957	–	–	–
*Nanorana xuelinensis* sp. nov.	Lancang, Yunnan, China	KIZL2019012	MZ410625	MZ410628	–	–	–
*Nanorana xuelinensis* sp. nov.	Lancang, Yunnan, China	KIZL2019013	MZ410624	MZ410627	–	–	–
*Nanorana xuelinensis* sp. nov.	Lancang, Yunnan, China	KIZL2019014	MZ410623	MZ410626	–	–	–
*Limnonectes fragilis*	Hainan, China	ZNAC11006	AY899241	AY899241	–	–	–
*Quasipaa boulengeri*	Yichang, Hubei, China	KIZ-HUB292	KX645665	KX645665	–	–	–

### Phylogenetic analyses

Total genomic DNA was isolated from the tissue samples of three individuals. *Quasipaa boulengeri* (Günther, 1889) and *Limnonectes fragilis* (Liu & Hu, 1973) were used as outgroups according to Qi et al. (2019). The mitochondrial genes 12S ribosomal RNA (12S) and 16S ribosomal RNA (16S), and the nuclear genes recombination activating protein 1 (Rag1), rhodopsin (Rhod), and tyrosinase (Tyr) of 19 known *Nanorana* species and two outgroup species were obtained from GenBank. Detail information of these materials are given in Table 1.

Sequences were aligned using ClustalW (Thompson et al. 1994) integrated in MEGA X (Kumar et al. 2018) with default parameters. The genetic divergences (uncorrected *p*-distance) were calculated in MEGA X (Kumar et al. 2018). 12S, 16S, Rag1, Rhod, and Tyr gene segments were concatenated seriatim into a single partition. Bayesian inference (BI) was performed in MrBayes 3.2.7 (Ronquist et al. 2012) and used the Akaike information criterion (AIC) in ModelFinder (Kalyaanamoorthy et al. 2017) to calculate that GTR+F+I+G4 was the best-fit model of evolution for 12S and 16S; HKY+F+I was the best-fit model of evolution for Rag1, Rhod, and Tyr. Two runs were performed simultaneously with four Markov chains starting from a random tree. The chains were run for 1,000,000 generations and sampled every 100 generations. The first 25% of the sampled trees was discarded as burn-in after the standard deviation of split frequencies of the two runs was less than a value of 0.01, and then the remaining trees were used to create a 50% majority-rule consensus tree and to estimate Bayesian posterior probabilities. Maximum likelihood (ML) analysis was performed in IQ-TREE (Nguyen et al. 2015) and used the Akaike information criterion (AIC) in ModelFinder (Kalyaanamoorthy et al. 2017) to calculate that GTR+F+R3 was the best-fit model of evolution for 12S and 16S, and that TPM3+F+I was the best-fit model for Rag1, Rhod, and Tyr. 1000 bootstrap pseudoreplicates via the ultrafast bootstrap (UFB; Hoang et al. 2018) approximation algorithm were used to construct a final consensus tree.

### Morphology

All measurements were taken with digital calipers to the nearest 0.1 mm. Morphological characters used and their measurement methods followed Qi et al. (2019). The morphometrics and character terminology include:

**AG** axilla to groin, distance from posterior base of forelimb at its emergence from body to anterior base of hindlimb at its emergence from body;

**EHD** eye horizontal diameter;

**END** eye to nostril distance, distance from anterior corner of eye to nostril;

**FL** foot length, from proximal end of inner metatarsal tubercle to tip of toe IV;

**FML** femur length;

**HAL** hand length, from proximal end of outer metacarpal tubercle to tip of the finger III;

**HH** head height, greatest height of head;

**HL** head length, from posterior corner of mandible to tip of snout;

**HW** head width, at the greatest cranial width;

**ID** internasal distance, distance between nostrils;

**IOD** interorbital distance, least distance between upper eyelids;

**LAD** diameter of lower arm;

**LAL** length of lower arm, from proximal end of outer metacarpal tubercle to elbow joint;

**SL** snout length, from tip of snout to the anterior corner of eye distance;

**SND** snout to nostril distance, distance from tip of snout to nostril;

**SVL** Snout–vent length, from tip of snout to vent;

**TDH** horizontal diameter of tympanum;

**TDV** vertical diameter of tympanum;

**TFL** length of tarsus and foot, from proximal end of tarsus to tip of the toe IV;

**TIL** tibia length;

**UEW** upper eyelid width, maximum width of upper eyelid.

All measurements were taken on the left side of the examined specimen. It should be noted that because the limbs of our specimens cannot be spread, the characters FLL (length of forelimb, from axilla to tip of finger III) and HLL (length of hindlimb, from tip of disk of toe IV to vent) in Qi et al. (2019) are not provided here.

## Results

### Genealogical relationships

**Figure 1.**
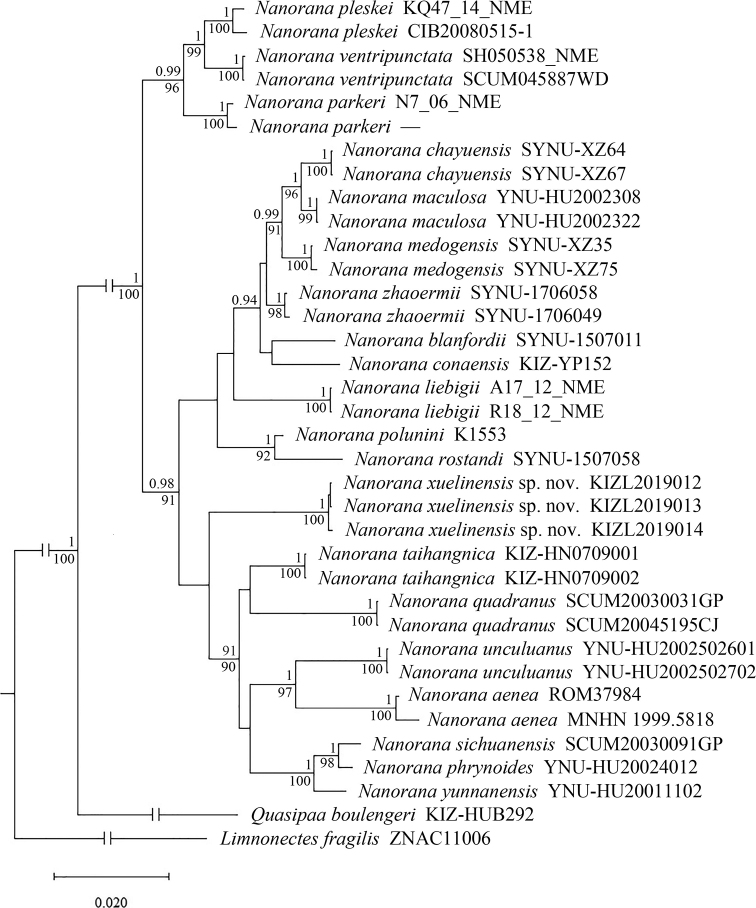
Bayesian inference tree of the genus *Nanorana* based on the sequences of the mitochondrial 12S and 16S, and the nuclear Rag1, Rhod, and Tyr gene. *Limnonectes fragilis* and *Quasipaa boulengeri* were included as outgroup. Numbers above branches indicate Bayesian posterior probabilities (≥0.9 retained) and numbers below branches indicate the ML ultrafast bootstrap (UFB) (≥90 retained).

The results of BI and ML phylogenetic trees were constructed based on the concatenated DNA sequences and resulted in approximately identical topologies (Fig. 1). The phylogenetic tree showed that the newly discovered population from Xuelin Township, Lancang County is a member of *Nanorana*; however, its phylogenetic position in the genus was not clearly resolved. The newly discovered population formed a unique clade sister to the clade consisting of *Nanorana aenea* (Smith, 1922), *N. phrynoides* (Boulenger, 1917), *N. quadranus* (Liu, Hu & Yang, 1960), *N. sichuanensis* (Dubois, 1987), *N. taihangnica* (Chen & Jiang, 2002), *N. unculuanus* (Liu, Hu & Yang, 1960), and *N. yunnanensis* (Anderson, 1879), but the node supports were very low.

### Genetic distances

The uncorrected *p*-distances calculated from 12S rRNA and 16S rRNA gene fragment sequences of the examined species are shown in Tables 2 and 3, respectively. The observed distances calculated from 12S gene between the sequences of the specimens collected from Xuelin Township, Lancang County and the homologous sequences obtained from GenBank ranged from 3.9% to 7.6%. The observed distances calculated from 16S gene between the sequences of the specimens collected from Xuelin Township, Lancang County and the homologous sequences obtained from GenBank ranged from 5.2% to 7.3%.

**Table 2.** Mean uncorrected pairwise genetic distances (%) among the species of *Nanorana* and outgroups based on partial 12S gene.

	1	2	3	4	5	6	7	8	9	10	11	12	13	14	15	16	17	18	19	20
1 *Nanorana aenea*																				
2 *N. blanfordii*	7.8																			
3 *N. chayuensis*	6.9	5.0																		
4 *N. cuonaensis*	7.7	5.3	6.6																	
5 *N. liebigii*	6.9	5.8	5.6	6.3																
6 *N. maculosa*	7.2	4.8	2.0	5.8	4.9															
7 *N. medogensis*	6.1	3.9	3.3	4.0	4.5	2.8														
8 *N. parkeri*	6.8	6.9	5.3	7.1	5.9	4.9	4.5													
9 *N. phrynoides*	6.6	7.3	7.1	8.3	7.3	7.1	6.0	8.8												
10 *N. pleskei*	6.0	5.1	5.3	6.7	5.7	5.3	4.8	3.6	7.6											
11 *N. quadranus*	7.5	8.1	7.2	8.1	7.4	7.9	7.0	8.4	7.9	7.6										
12 *N. rostandi*	6.1	6.3	5.5	7.5	5.6	5.8	5.4	5.9	7.3	5.5	7.6									
13 *N. sichuanensis*	6.8	7.5	7.3	9.5	7.3	7.4	6.7	9.1	1.2	7.8	8.1	7.5								
14 *N. taihangnica*	5.2	5.3	5.1	6.3	4.4	4.9	4.0	6.1	5.4	4.6	5.4	5.3	5.6							
15 *N. unculuanus*	5.5	5.0	5.4	7.1	5.4	5.2	4.3	7.3	5.4	5.9	7.4	4.8	6.1	3.7						
16 *N. ventripundata*	8.4	6.3	6.7	8.3	6.4	6.6	6.3	5.4	8.2	3.4	8.6	7.4	8.9	6.7	6.7					
17 *N. yunnanensis*	4.5	6.5	5.2	8.1	6.8	5.7	5.6	6.0	1.9	5.2	7.4	5.7	2.2	4.9	5.4	6.6				
18 *N. zhaoermii*	6.0	3.2	3.2	3.5	4.1	2.1	1.4	5.0	5.9	4.1	6.3	4.4	6.8	3.5	3.5	5.1	5.8			
19 *Nanorana xuelinensis* sp. nov.	6.3	5.6	6.0	6.6	4.0	5.4	4.6	5.9	6.8	6.0	7.1	6.0	7.1	3.9	6.1	7.6	6.5	4.5		
20 *Quasipaa boulengeri*	11.9	12.8	12.5	14.1	11.8	12.4	12.5	12.8	14.7	12.7	13.5	11.9	14.9	12.0	12.9	14.8	13.6	14.0	11.8	
21 *Limnonectes fragilis*	18.1	16.0	17.8	20.0	17.5	18.0	17.8	17.8	18.5	17.0	18.7	18.4	17.5	17.5	18.0	19.1	17.3	18.0	17.4	15.9

**Table 3.** Mean uncorrected pairwise genetic distances (%) among the species of *Nanorana* and outgroups based on partial 16S gene.

	1	2	3	4	5	6	7	8	9	10	11	12	13	14	15	16	17	18	19	20	21
1 *Nanorana aenea*																					
2 *N. blanfordii*	5.1																				
3 *N. chayuensis*	5.2	2.4																			
4 *N. cuonaensis*	5.5	2.8	3.6																		
5 *N. liebigii*	6.2	4.1	4.1	5.3																	
6 *N. maculosa*	5.4	2.5	0.9	4.0	3.8																
7 *N. medogensis*	5.9	3.0	2.5	4.4	4.0	2.2															
8 *N. parkeri*	5.5	2.9	3.3	3.5	4.4	3.2	3.8														
9 *N. phrynoides*	4.7	4.5	5.3	4.0	4.6	5.3	5.4	4.4													
10 *N. pleskei*	5.2	2.9	4.1	4.4	4.3	3.6	4.3	3.1	4.7												
11 *N. polunini*	6.0	3.6	4.1	4.4	6.3	4.1	3.8	4.1	6.4	5.2											
12 *N. quadranus*	6.1	4.2	5.8	5.7	6.8	5.8	5.3	5.5	5.1	6.1	6.2										
13 *N. rostandi*	6.6	3.4	4.5	4.1	5.3	4.3	4.3	5.1	5.8	5.3	2.3	5.5									
14 *N. sichuanensis*	5.2	4.4	5.5	4.2	5.3	5.5	5.6	4.5	1.1	4.2	6.6	4.5	5.6								
15 *N. taihangnica*	4.6	3.2	4.5	3.6	4.4	4.2	4.2	3.0	3.8	3.1	5.1	6.3	4.9	4.0							
16 *N. unculuanus*	4.6	5.3	6.1	6.6	6.4	6.2	6.1	5.9	6.0	5.7	8.3	6.9	6.8	5.8	5.4						
17 *N. ventripundata*	4.9	2.5	3.4	3.9	3.9	3.3	3.9	2.3	3.7	2.0	4.9	5.0	4.5	3.5	3.9	5.5					
18 *N. yunnanensis*	5.3	4.5	5.4	4.4	5.3	5.5	5.6	4.5	2.2	4.8	6.1	5.1	5.4	2.2	4.2	6.0	4.3				
19 *N. zhaoermii*	5.5	2.4	2.1	3.1	4.3	2.2	2.6	2.9	4.6	3.0	3.2	4.0	4.7	4.0	3.6	6.0	2.9	4.4			
20 *Nanorana xuelinensis* sp. nov.	6.7	5.8	5.8	5.6	6.5	5.4	5.2	6.0	5.6	6.0	6.6	6.6	5.9	6.2	5.3	7.3	5.7	6.2	5.3		
21 *Quasipaa boulengeri*	8.4	7.8	7.5	6.7	8.0	7.2	7.4	6.5	7.6	7.3	8.8	8.7	9.4	8.0	7.1	9.2	7.7	7.8	6.1	8.3	
22 *Limnonectes fragilis*	12.0	11.8	12.0	11.7	12.2	11.6	11.8	11.1	12.4	12.2	16.2	13.3	14.3	12.6	11.5	12.2	12.5	12.8	11.4	12.3	10.3

### Systematics

#### Nanorana xuelinensis sp. nov.

Taxon classificationAnimaliaAnuraDicroglossidae

23D2138E-2CA2-5FC4-95EE-6B9D27E6DCB1

http://zoobank.org/3BB0CC31-8B68-4EA7-BC7C-DDF7D78C977F

**Figure 2.**
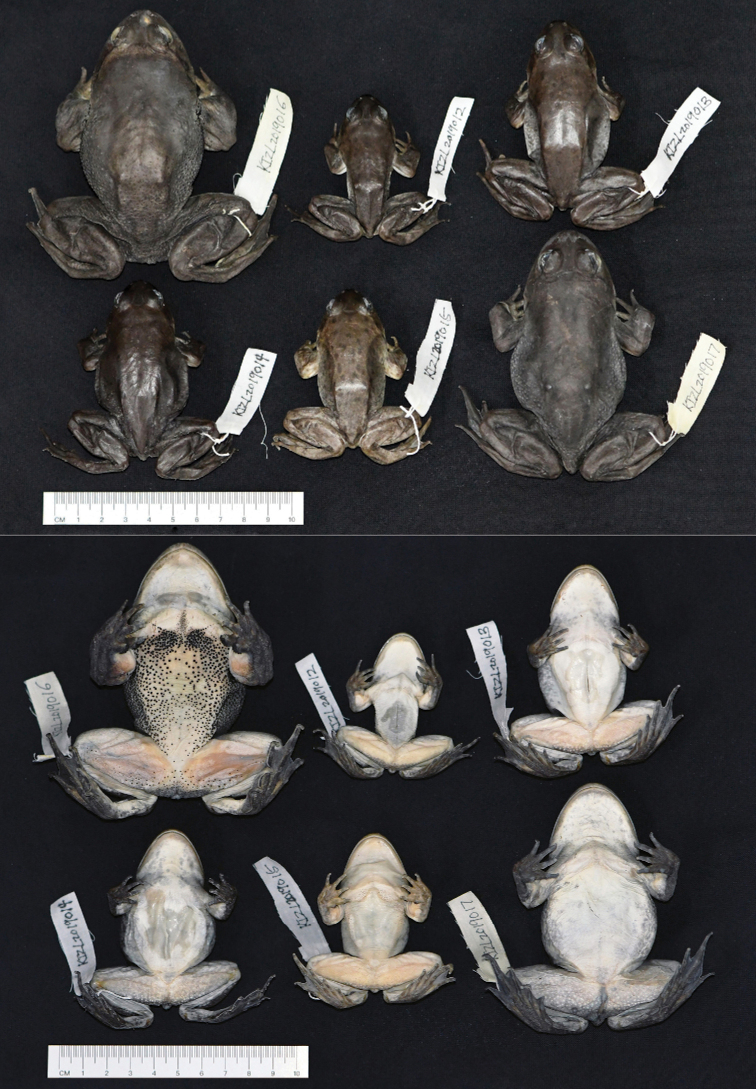
Dorsal and ventral views of the specimens of the type series of *Nanorana xuelinensis* sp. nov. in preservative.

**Figure 3.**
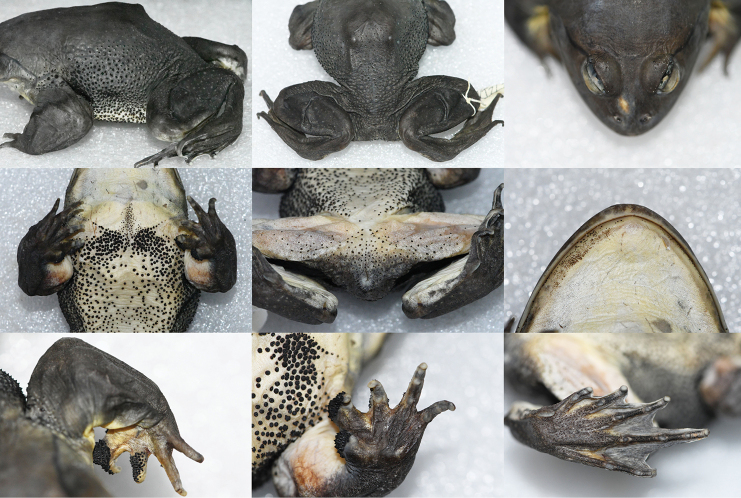
Various views of the male holotype (KIZL2019016) of *Nanorana xuelinensis* sp. nov. in preservative.

**Figure 4.**
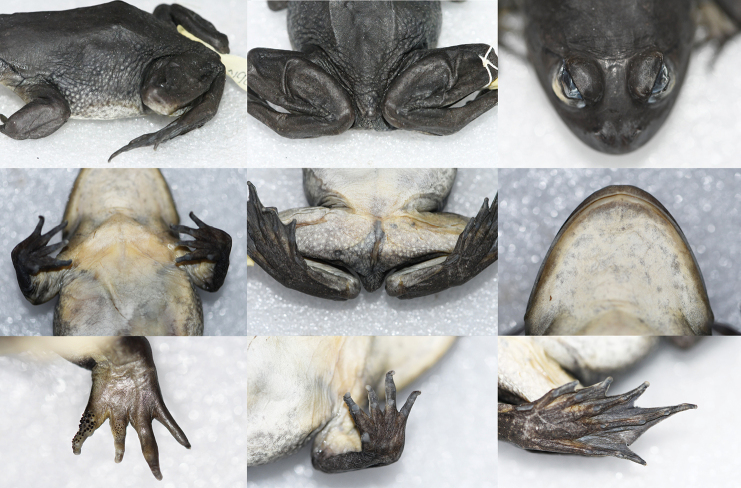
Various views of the female paratype (KIZL2019017) of *Nanorana xuelinensis* sp. nov. in preservative.

**Figure 5.**
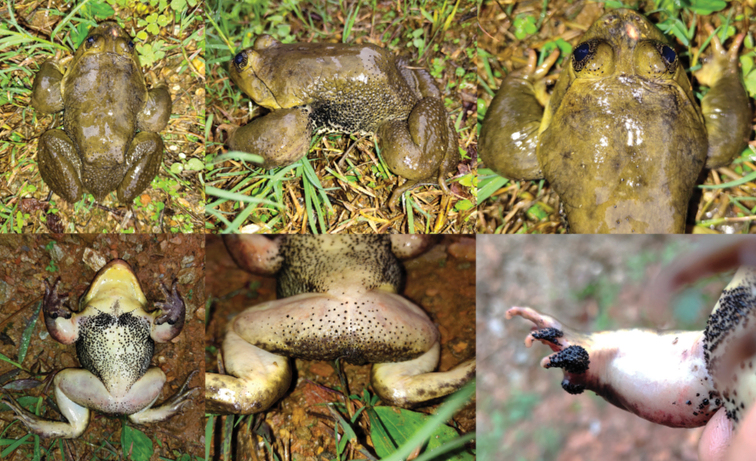
Different views of the male holotype (KIZL2019016) of *Nanorana xuelinensis* sp. nov. in life.

**Figure 6.**
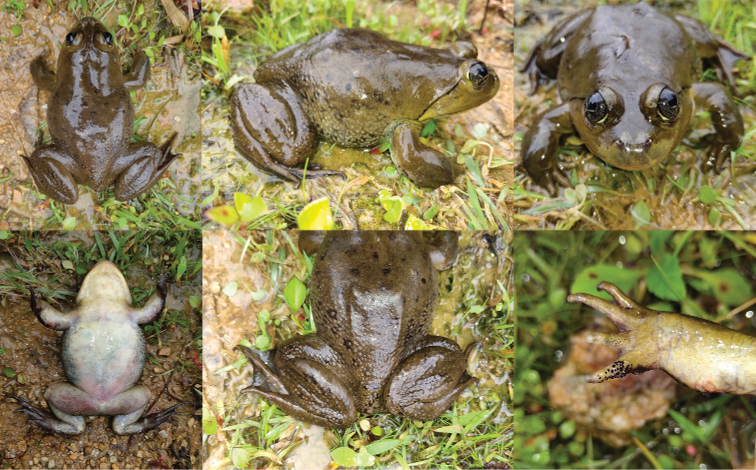
Different views of the female paratype (KIZL2019017) of *Nanorana xuelinensis* sp. nov. in life.

Figures 2 , 3 , 4 , 5 , 6

##### Holotype.

KIZL2019016, adult male, collected on 13 July 2019 by Shuo Liu from Xuelin Township, Lancang County, Puer City, Yunnan Province, China (23°2'38"N, 99°32'35"E; at an elevation of 1840 m asl).

##### Paratypes.

KIZL2019012 and KIZL2019015, two subadult males; KIZL2019013 and KIZL2019014, two subadult females; and KIZL2019017, adult female. All with same collection information as for the holotype.

##### Diagnosis.

Large body size, SVL 101.7–107.3 mm in adults; adult males with keratinized spines on chest, belly, lateral body, posterior dorsum, buttocks, outer side of the fore limbs, the inner metacarpal tubercle, fingers I and II, and upper eyelids; no spines on the inner side of the lower and upper arm; forelimbs strongly hypertrophied in adult males; tympanum big but indistinct, ca 2/3 of eye diameter; anterior dorsum skin smooth; dorsolateral folds absent; finger I longer than finger II; webbing deeply incurved between tips of toes; no tarsal fold; present outer metacarpal tubercle and absent outer metatarsal tubercle; vomerine teeth distinct.

The living specimens were yellowish brown with distinct or indistinct black spots on the dorsum and sides of the body and the dorsal side of limbs; no band on arms and legs. Ventral surface white with no spots, throat yellow in adult males.

##### Description of holotype.

Adult male, habitus very stout, SVL 107.3 mm, large size in genus *Nanorana*; head flat and broader than long (HL/HW 0.85, HH/HL 0.53); snout blunt and rounded in both dorsal and lateral views; canthus rostralis obtuse; tympanum large and very indistinct (TDH/EHD 0.76); supratympanic fold extending from eye over tympanum to shoulder; transversal fold behind eyes; eye relatively large (EHD/HL 0.26), pupil slightly rhombic; vomerine teeth distinct; tongue large and cordiform, deeply notched posteriorly.

Forelimbs short and strongly hypertrophied (LAD 18.8 mm); relative finger length: II < I < IV < III; inner metacarpal tubercle enlarged, dorsal surface of inner metacarpal tubercle, fingers I, and finger II with black keratinized nuptial spines, no spine on inner side of fore limbs, and a few spines on outer side of fore limbs; finger tips rounded but not dilated, fingers free, without webbing, no circum-marginal groove or lateroventral groove; subarticular tubercles distinct, outer metacarpal tubercle indistinct.

Hindlimbs rather long and stout; relative toe length: I < II < V < III < IV; tips of toes rounded but not dilated; subarticular tubercles oval and distinct, formula is 1, 1, 2, 3, 2; inner metatarsal tubercles elongated and pronounced; outer metatarsal tubercle absent; webbing deeply incurved between tips of toes, formula I 0-0- II 0-0- III 0--0- IV 0--0 V; lateral fringe on the outer side of toe V developed; no circum-marginal groove or lateroventral groove; tarsal fold absent.

**Figure 7.**
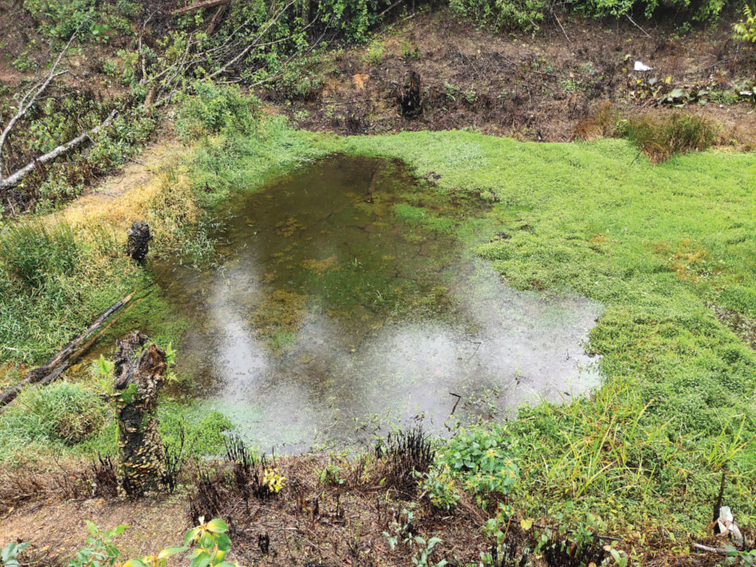
Habitat of *Nanorana xuelinensis* sp. nov. at the type locality.

Anterior dorsum skin smooth; keratinized spines present on chest, belly, lateral body, posterior dorsum, buttocks, and upper eyelids; spines most dense on axilla and each side of chest.

##### Coloration of holotype in life.

The coloration of dorsum is yellowish brown with very indistinct black spots in dorsum, and no band on arms and legs. Ventral surface white with no spots. The throat is yellow. The pupil is black, and the iris is light yellow with many black radial strips around the pupil.

##### Sexual dimorphism.

The forelimbs of adult males are strongly hypertrophied; in addition, adult males have keratinized spines on chest, belly, lateral body, posterior dorsum, buttocks, outer side of the fore limbs, the inner metacarpal tubercle, fingers I and II, and upper eyelids. The forelimbs of adult females are not hypertrophied, and adult females have distinct black spots on the dorsum, lateral body, and the dorsal side of limbs, no keratinized spines on chest, belly, lateral body, posterior dorsum, buttocks, and upper eyelids, and only some keratinized spines on finger I and a few small spines on finger II.

**Figure 8.**
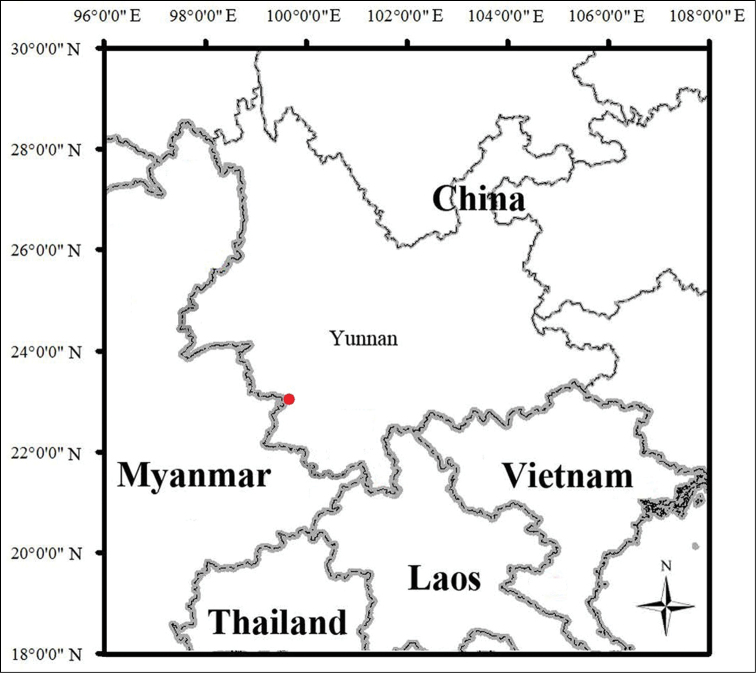
Map showing the type locality of *Nanorana xuelinensis* sp. nov. (red dot).

##### Etymology.

The name refers to Xuelin Township, the locality where the new species was found. We propose “Xuelin Paa Frog” or “Xuelin Spiny Frog” for the common English name and “雪林棘蛙” (Xuě Lín Jí Wā) for the common Chinese name of the new species.

**Table 4.** Morphological measurements (mm) of the type series of *Nanorana xuelinensis* sp. nov.

	KIZL2019016 Holotype Adult male	KIZL2019017 Paratype Adult female	KIZL2019012 Paratype Subadult male	KIZL2019013 Paratype Subadult female	KIZL2019014 Paratype Subadult female	KIZL2019015 Paratype Subadult male
SVL	107.3	101.7	60.3	79.2	75.1	66.9
AG	36.2	40.6	15.9	29.1	29.7	20.1
HL	35.9	36.4	23.7	27.0	27.7	25.8
HW	42.1	38.1	23.6	28.8	28.9	27.0
HH	19.1	18.9	11.6	14.1	15.4	13.1
SL	16.4	14.4	9.7	11.3	11.6	11.2
ID	7.3	7.4	4.6	5.7	5.6	5.5
IOD	4.1	4.7	2.5	3.3	3.9	3.3
UEW	7.0	7.2	4.3	5.6	5.7	5.1
EHD	9.5	10.8	6.6	8.4	8.3	8.4
TDH	7.2	7.7	4.5	5.6	5.7	4.6
TDV	6.6	4.8	3.5	4.4	4.2	3.6
SND	8.4	7.1	4.5	6.4	5.1	4.9
END	7.9	7.3	4.6	5.6	5.8	5.4
LAl	22.5	19.9	11.7	15.3	13.9	14.1
LAD	18.8	10.3	7.9	8.8	8.2	9.4
HAL	22.7	19.0	14.6	17.8	15.9	15.6
FML	45.4	41.8	26.8	34.6	33.5	29.5
TIL	42.5	39.2	25.7	33.2	31.1	28.9
TFL	65.9	63.1	41.9	54.2	49.9	45.8
FL	44.3	42.7	29.1	36.0	33.6	33.1

##### Distribution.

*Nanorana xuelinensis* sp. nov. is recorded in Lancang County (Pu’er City), Shuangjiang County (Lincang City), and Jinghong City (Xishuangbanna Prefecture), Yunnan Province, China.

##### Habitat.

The type series was found in a still-water pond. At the type locality we found three other species of amphibians: Chirixalus cf. doriae Boulenger, 1893; *Raorchestes hillisi* Jiang Ren, Guo, Wang & Li, 2020; *Tylototriton verrucosus* Anderson, 1871a; and three species of reptiles: *Calotes emma* Gray, 1845; *Pareas xuelinensis* Liu & Rao, 2021; and *Pseudocalotes microlepis* (Boulenger, 1887).

##### Comparisons.

*Nanorana xuelinensis* sp. nov. differs from *N. aenea*, *N. annandalii* (Boulenger, 1920), *N. gammii* (Anderson, 1871b), *N. liebigii* (Günther, 1860), *N. polunini* (Smith, 1951), *N. rarica* (Dubois, Matsui & Ohler, 2001), *N. rostandi* (Dubois, 1974), and *N. unculuanus* by the absence of dorsolateral fold (vs presence).

*Nanorana xuelinensis* sp. nov. differs from *N. arnoldi* (Dubois, 1975), *N. maculosa* (Liu, Hu & Yang, 1960), *N. yunnanensis*, and *N. zhaoermii* Qi, Zhou, Lu & Li, 2019 by the spines present only on finger I and finger II in adult males (vs present on finger I–III).

*Nanorana xuelinensis* sp. nov. differs from *N. arunachalensis* (Saikia, Sinha & Kharkongor, 2017), *N. blanfordii* (Boulenger, 1882), *N. chayuensis* (Ye, 1977), *N. conaensis* (Fei & Huang, 1981), *N. minica* (Dubois, 1975), and *N. mokokchungensis* (Das & Chanda, 2000) by its larger body size.

*Nanorana xuelinensis* sp. nov. differs from *N. feae* (Boulenger, 1887) by the absence of spines on the inner side of the fore limbs in adult males (vs. presence).

*Nanorana xuelinensis* sp. nov. differs from *N. kangxianensis* (Yang, Wang, Hu & Jiang, 2011), *N. quadranus*, *N. taihangensis* by the strongly hypertrophied forelimbs in adult males (vs not hypertrophied), and by the presence of nuptial spines on the chest and fingers in adult males (vs absence).

*Nanorana xuelinensis* sp. nov. differs from *N. medogensis* (Fei & Ye, 1999), *N. phrynoides*, and *N. sichuanensis* by smooth anterior dorsum skin (vs many warts present).

*Nanorana xuelinensis* sp. nov. differs from *N. parkeri* (Stejneger, 1927), *N. pleskei* Günther, 1896, and *N. ventripunctata* Fei & Huang, 1985 by the shape of the nuptial spines (large and conical spines vs tiny and compact spines).

*Nanorana xuelinensis* sp. nov. differs from *N. vicina* (Stoliczka, 1872) by its toes ca 2/3 webbed (vs fully webbed) and by the absence of bands on the hind limbs (vs presence).

## Discussion

Most species of *Nanorana* live in running waters, especially in swiftly running waters (Dubois and Ohler 2005; Ohler and Dubois 2006) such as rivers or streams, except for *N. parkeri*, *N. pleskei*, and *N. ventripunctata*, which have produced a series of specialized adaptations to high-altitude habitats (Che et al. 2020). However, the habitat of the *Nanorana xuelinensis* sp. nov. is distinctive. All specimens of *Nanorana xuelinensis* sp. nov. were found in still waters in different seasons. Why this species lives in still waters needs further study.

Morphologically, *Nanorana xuelinensis* sp. nov. is obviously different from all other known species of the genus *Nanorana*. The skins of most species of *Nanorana* are rough with more or less tubercles or warts (Che et al. 2020). However, the skin of *Nanorana xuelinensis* sp. nov. is quite smooth on most areas of the body. Most males of the tribe Paini have spines on the fingers, arms, or breast. The presence of these spines is an adaptation to breeding in swiftly running waters, helping the males grasp of the females (Ohler and Dubois 2006). Although *Nanorana xuelinensis* sp. nov. does not live in running waters, the males still may need spines to help grasp females due to their smoother skins. But why the males of *Nanorana xuelinensis* sp. nov. have so many keratinized spines on the other areas of the body except for the fingers and breast we do not yet know, and the reason for this feature also needs further study.

The genus *Nanorana* contains 30 species, of which 22 species are recorded in China (Frost 2021); however, *N. arnoldi* is not recorded from China according to AmphibiaChina (2021), which lists only 21 species. This is probably due to an erroneous synonymy: *N. chayuensis* was placed into the synonymy of *N. arnoldi* by Dubois (1980), which subsequently was rejected by Hu (1985). In the phylogenetic analyses of Che et al. (2009), the gene sequences of *N. arnoldi* and *N. chayuensis* clustered together, but these sequences of *N. arnoldi* were from Yunnan, China, which were possibly wrongly identified and probably belong to *N. chayuensis*. We speculate that the true *N. arnoldi* is distributed in northern Myanmar, and not in China. Because we do not have specimens from northern Myanmar, whether *N. chayuensis* and *N. arnoldi* are the same species remains to be solved, but for the time being, we support AmphibiaChina (2021) in treating *N. chayuensis* as valid and considering that *N. arnoldi* is not distributed in China. Further collections from both countries will clarify this taxonomic conundrum.

## Supplementary Material

XML Treatment for Nanorana xuelinensis

## References

[B1] AmphibiaChina (2021) The database of Chinese amphibians. Electronic Database. http://www.amphibiachina.org [accessed on 6 March 2021]

[B2] Anderson J (1871a) Description of a new genus of newts from western Yunan. Proceedings of the Zoological Society of London 1871: 423–425.

[B3] Anderson J (1871b) A list of the reptilian accession to the Indian Museum, Calcutta from 1865 to 1870, with a description of some new species. Journal of the Asiatic Society of Bengal 40: 12–39.

[B4] Anderson J (1879) Anatomical and Zoological Researches: Comprising an Account of the Zoological Results of the Two Expeditions to Western Yunnan in 1868 and 1875; and a Monograph of the Two Cetacean Genera Platanista and Orcella. 2 Volumes. Bernard Quaritch, London, 985 pp. 10.5962/bhl.title.55401

[B5] Boulenger GA (1882) Catalogue of the BatrachiaSalientia s. Caudata in the Collections of the British Museum. (Natural History). 2^nd^ edn. Taylor and Francis, London, 503 pp.

[B6] Boulenger GA (1887) An account of the batrachians obtained in Burma by M.L. Fea of the Genoa Civic Museum. Annali del Museo Civico di Storia Naturale di Genova 5: 418–424.

[B7] Boulenger GA (1888) An account of the reptiles and batrachians obtained in Tenasserim by M. L. Fea, of the Genova Civic Museum. Annali del Museo Civico di Storia Naturale di Genova 5: 474–486.

[B8] Boulenger GA (1893) Concluding report on the reptiles and batrachians obtained in Burma by Signor L. Fea dealing with the collection made in Pegu and the Karin Hills in 1887–88. Annali del Museo Civico di Storia Naturale di Genova 13: 304–347.

[B9] Boulenger GA (1917) Descriptions of new frogs of the genus *Rana*. Annals and Magazine of Natural History (Series 8) 20: 413–418. 10.1080/00222931709487029

[B10] Boulenger GA (1920) A monograph of the South Asian, Papuan, Melanesian and Australian frogs of the genus *Rana*. Records of the Indian Museum 20: 1–226. 10.5962/bhl.title.12471

[B11] Bourret R (1939) Notes herpétologiques sur l’Indochine française. XVII. Reptiles et batraciens reçus au Laboratoire des Sciences Naturelles de l’Université au cors de l’année 1938. Descriptions de trois espèces nouvelles. Annexe au Bulletin Général de l’Instruction Publique. Hanoi 1939: 13–34.

[B12] Che J Hu JS Zhou WW Murphy RW Papenfuss TJ Chen MY Rao DQ Li PP Zhang YP (2009) Phylogeny of the Asian spiny frog tribe Paini (Family Dicroglossidae) sensu Dubois. Molecular Phylogenetics and Evolution 50: 59–73. 10.1016/j.ympev.2008.10.007 18992827

[B13] Che J Zhou WW Hu JS Yan F Papenfuss TJ Wake DB Zhang YP (2010) Spiny frogs (Paini) illuminate the history of the Himalayan region and Southeast Asia. Proceedings of the National Academy of Sciences 107: 13765–13770. 10.1073/pnas.1008415107 PMC2922240 20643945

[B14] Che J Jiang K Yan F Zhang YP (2020) Amphibians and Reptiles in Tibet – Diversity and Evolution. Science Press, Beijing, 803 pp.

[B15] Chen LQ Murphy RW Lathrop A Ngo A Orlov NL Ho CT Somorjai ILM (2005) Taxonomic chaos in Asian ranid frogs: an initial phylogenetic resolution. Herpetological Journal 15: 231–243.

[B16] Chen XH Jiang JP (2002) A new species of the genus *Paa* from China. Herpetologica Sinica 9: 231.

[B17] Das I Chanda SK (2000) A new species of *Scutiger* (Anura: Megophryidae) from Nagaland, north-eastern India. Herpetological Journal 10: 69–72.

[B18] Dubois A (1974) Diagnoses de trois espèces Nouvelles d’amphibiens du Népal. Bulletin de la Société Zoologique de France 98: 495–497.

[B19] Dubois A (1975) Un nouveau sous-genre (*Paa*) et trois nouvelles espèces du genre *Rana*. Remarques sur la phylogenies des ranidés (Amphibiens, Anoures). Bulletin du Museum National d’Histoire Naturelle 324: 1093–1115.

[B20] Dubois A (1980) Notes sur la systématique et la répartition des amphibiens anoures de Chine et des régions avoisinantes. 3. *Rana maculata* Liu, Hu & Yang, 1970, Rana (Paa) arnoldi Dubois, 1975 et *Rana maculosa chayuensis* Ye, 1977. Bulletin Mensuel de la Société Linnéenne de Lyon 49: 142–147. 10.3406/linly.1980.10415

[B21] Dubois A (1987) Miscellanea taxinomica batrachologica (I). Alytes 5: 7–95.

[B22] Dubois A (1992) Notes sur la classification des Ranidae (Amphibiens, Anoures). Bulletin mensuel de la Société linnéenne de Lyon 61: 305–352. 10.3406/linly.1992.11011

[B23] Dubois A Matsui M Ohler A (2001) A replacement name for Rana (Paa) rara Dubois & Matsui, 1983 (Amphibia, Anura, Ranidae, Raninae). Alytes 19: 2–4. 10.2307/1445091

[B24] Dubois A Ohler A (2005) Taxonomic notes on the Asian frogs of the tribe Paini (Ranidae, Dicroglossinae): 1. Morphology and synonymy of *Chaparana aenea* (Smith, 1922), with proposal of a new statistical method for testing homogeneity of small samples. Journal of Natural History 39: 1759–1778. 10.1080/00222930400023735

[B25] Dubois A Ohler A Pyron RA (2021) New concepts and methods for phylogenetic taxonomy and nomenclature in zoology, exemplified by a new ranked cladonomy of Recent amphibians (Lissamphibia). Megataxa 5: 1–738. 10.11646/megataxa.5.1.1

[B26] Fei L (1999) Atlas of Amphibians of China. Henan Press of Science and Technology, Zhengzhou, 432 pp.

[B27] Fei L Huang YZ (1985) A new species of the genus *Nanorana* (Amphibia: Ranidae) from northwestern Yunnan, China. Acta Biologica Plateau Sinica 4: 71–75.

[B28] Frost DR (2021) Amphibian Species of the World: an Online Reference. Version 6.1. Electronic Database. https://amphibiansoftheworld.amnh.org/index.php [accessed on 6 March 2021]

[B29] Gray JE (1845) Catalogue of the Specimens of Lizards in the Collection of the British Museum. Trustees of the British Museum/Edward Newman, London, 289 pp.

[B30] Günther ACLG (1860) Contribution to the knowledge of the reptiles of the Himalaya mountains. Proceedings of the Zoological Society of London 1860: 148–175.

[B31] Günther ACLG (1889) Third contribution to our knowledge of reptiles and fishes from the upper Yangtze-Kiang. Annals and Magazine of Natural History 4: 218–229. 10.1080/00222938909460506

[B32] Günther ACLG (1896) Report on the collections of reptiles, batrachians and fishes made by Messrs Potanin and Berezowski in the Chinese provinces Kansu and Sze-chuen. Annuaire du Musée Zoologique de l’Academie Impériale des Sciences de St. Pétersbourg 1: 199–219.

[B33] Hoang DT Chernomor O von Haeseler A Minh BQ Vinh LS (2018) UFBoot2: improving the ultrafast bootstrap approximation. Molecular Biology and Evolution 35: 518–522. 10.1093/molbev/msx281 29077904 PMC5850222

[B34] Hu SQ (1985) Raninae (China). In: Frost DR (Ed.) Amphibian Species of the World: A Taxonomic and Geographical Reference.Allen Press, Kansas, 451–521.

[B35] Huang YZ Fei L (1981) Two new species of amphibians from Xizang. Acta Zootaxonomica Sinica 6: 211–215.

[B36] Jiang JP Dubois A Ohler A Tillier A Chen XH Xie F Stöck M (2005) Phylogenetic relationships of the tribe Paini (Amphibia, Anura, Ranidae) based on partial sequences of mitochondrial 12S and 16S rRNA genes. Zoological Science 22: 353–362. 10.2108/zsj.22.353 15795498

[B37] Jiang K Ren JL Wang J Guo JF Wang Z Liu YH Jiang DC Li JT (2020) Taxonomic revision of *Raorchestes menglaensis* (Kou, 1990) (Amphibia: Anura), with descriptions of two new species from Yunnan, China. Asian Herpetological Research 11: 263–281. 10.16373/j.cnki.ahr.200018

[B38] Kalyaanamoorthy S Minh BQ Wong TKF von Haeseler A Jermiin LS (2017) ModelFinder: fast model selection for accurate phylogenetic estimates. Nature Methods 14: 587–589. 10.1038/nmeth.4285 28481363 PMC5453245

[B39] Kumar S Stecher G Li M Knyaz C Tamura K (2018) MEGA X: Molecular Evolutionary Genetics Analysis across computing platforms. Molecular Biology and Evolution 35: 1547–1549. 10.1093/molbev/msy096 29722887 PMC5967553

[B40] Liu CZ Hu SQ Fei L Huang CC (1973) On collections of amphibians from Hainan Island. Acta Zoologica Sinica 19: 385–404.

[B41] Liu CZ Hu SQ Yang FH (1960) Amphibia of Yunnan collected in 1958. Acta Zoologica Sinica 12: 149–174.

[B42] Liu S Rao DQ (2021) A new species of the genus *Pareas* (Squamata, Pareidae) from Yunnan, China. ZooKeys 1011: 121–138. 10.3897/zookeys.1011.59029 33564273 PMC7843430

[B43] Nguyen LT Schmidt HA von Haeseler A Minh BQ (2015) IQ-TREE: a fast and effective stochastic algorithm for estimating maximum-likelihood phylogenies. Molecular Biology and Evolution 32: 268–274. 10.1093/molbev/msu300 25371430 PMC4271533

[B44] Ohler A Dubois A (2006) Phylogenetic relationships and generic taxonomy of the tribe Paini (Amphibia, Anura, Ranidae, Dicroglossinae), with diagnoses of two new genera. Zoosystema 28: 769–784.

[B45] Palumbi SR Martin A Romano S McMillan W Stice L Grabowski G (1991) The Simple Fool’s Guide to PCR. University of Hawaii Press, Honolulu, 94 pp.

[B46] Qi S Zhou ZY Lu YY Li JL Qin HH Hou M Zhang Y Ma JZ Li PP (2019) A new species of *Nanorana* (Anura: Dicroglossidae) from southern Tibet, China. Russian Journal of Herpetology 26: 159–174. 10.30906/1026-2296-2019-26-3-159-174

[B47] Roelants K Jiang JP Bossuyt F (2004) Endemic ranid (Amphibia: Anura) genera in southern mountain ranges of the Indian subcontinent represent ancient frog lineages: evidence from molecular data. Molecular Phylogenetics and Evolution 31: 730–740. 10.1016/j.ympev.2003.09.011 15062806

[B48] Ronquist F Teslenko M Van Der Mark P Ayres DL Darling A Höhna S Larget B Liu L Suchard MA Huelsenbeck JP (2012) MrBayes 3.2: efficient Bayesian phylogenetic inference and model choice across a large model space. Systematic Biology 61: 539–542. 10.1093/sysbio/sys029 22357727 PMC3329765

[B49] Saikia B Sinha B Kharkongor I (2017) *Odorrana arunachalensis*: a new species of Cascade Frog (Anura: Ranidae) from Talle Valley Wildlife Sanctuary, Arunachal Pradesh, India. Journal of Bioresources 4: 30–41.

[B50] Sichuan Institute of Biology Herpetology Department (1977) A survey of amphibians in Xizang (Tibet). Acta Zoologica Sinica 23: 54–63.

[B51] Smith MA (1922) Notes on reptiles and batrachians from Siam and Indo-China (no. 1). Journal of the Natural History Society of Siam 4: 203–214.

[B52] Smith MA (1951) On a collection of amphibians and reptiles from Nepal. Annals and Magazine of Natural History (Series 12) 6: 726–728. 10.1080/00222935308654472

[B53] Stejneger L (1927) A new genus and species of frog from Tibet. Journal of the Washington Academy of Sciences 17: 317–319.

[B54] Stoliczka F (1872) Notes on some new species of Reptilia and Amphibia, collected by Dr. W. Waagen in north-western Punjab. Proceedings of the Asiatic Society of Bengal 1872: 124–131.

[B55] Thompson JD Higgins DG Gibson TJ (1994) CLUSTAL W: improving the sensitivity of progressive multiple sequence alignment through sequence weighting, position-specific gap penalties and weight matrix choice. Nucleic Acids Research 22: 4673–4680. 10.1093/nar/22.22.4673 7984417 PMC308517

[B56] Yang X Wang B Hu JH Jiang JP (2011) A new species of the genus *Feirana* (Amphibia: Anura: Dicroglossidae) from the western Qinling Mountains of China. Asian Herpetological Research 2: 72–86. 10.3724/SP.J.1245.2011.00072

